# Photostimulation of locus coeruleus CA1 catecholaminergic terminals reversed Spatial memory impairment in an alzheimer’s disease mouse model

**DOI:** 10.1007/s00213-025-06885-w

**Published:** 2025-09-08

**Authors:** Donovan K. Gálvez-Márquez, Oscar Urrego-Morales, Luis F. Rodríguez-Durán, Federico Bermudez-Rattoni

**Affiliations:** División de Neurociencias, Instituto de Fisiología Celular, Universidad Nacional Autónoma de México, Ciudad Universitaria, Mexico City, 04510 Mexico

**Keywords:** Dopamine, Alzheimer`s disease, Locus coeruleus, Hippocampus, Cognitive impairment

## Abstract

**Rationale:**

One of the earliest changes associated with Alzheimer’s disease (AD) is the loss of catecholaminergic terminals in the cortex and hippocampus originating from the Locus Coeruleus (LC). This decline leads to reduced catecholaminergic neurotransmitters in the hippocampus, affecting synaptic plasticity and spatial memory. However, it is unclear whether restoring catecholaminergic transmission in the terminals from the LC may alleviate the spatial memory deficits associated with AD.

**Objectives:**

This study aims to investigate the effects of optogenetic stimulation of LC catecholaminergic projections on alleviating spatial memory and synaptic plasticity deficits associated with AD.

**Methods:**

We conducted experiments using a 12-month-old 3xTgAD mouse model (AD-TH) that expresses Cre recombinase under the control of the tyrosine hydroxylase (TH) gene. This model enabled us to photostimulate the terminals from the LC in the hippocampal CA1 region before performing two different spatial memory tasks and inducing long-term plasticity.

**Results:**

Optogenetic stimulation successfully reversed the impairment of spatial memory retrieval in aging AD-TH mice. Furthermore, this stimulation restored levels of catecholaminergic neurotransmitters in the hippocampus and enhanced synaptic plasticity, as demonstrated by a long-term potentiation (LTP) protocol.

**Conclusions:**

These findings suggest a critical role for the LC-hippocampal CA1 catecholaminergic circuitry in disrupting synaptic plasticity and the spatial memory deficits characteristic of the early stages of AD. The study highlights the potential for targeting LC catecholaminergic pathways as a therapeutic strategy to improve cognitive deficits experienced by AD patients.

**Supplementary Information:**

The online version contains supplementary material available at 10.1007/s00213-025-06885-w.

##  Introduction

Alzheimer’s disease (AD) is characterized by the accumulation of amyloid beta (Aβ) peptides in extracellular aggregates and the formation of intracellular neurofibrillary tangles composed of Tau protein (O’Brien and Wong 2011; Sadigh-Eteghad et al. 2015; Zhang et al. 2018; Hampel et al. 2021). The build-up of Aβ peptide and hyperphosphorylated Tau protein tangles in different brain regions has been shown to disrupt the neurotransmitter system, synaptic plasticity, and cognitive function in laboratory and clinical studies (Tolar et al. 2021; Takahashi et al. 2021; Gloria et al. 2021; Wang et al. 2024). Despite these indicators of AD, a definitive primary cellular or molecular mechanism that explains the origin of the disease has yet to be identified (Ratan et al. 2023). Furthermore, extensive efforts to address these pathological features through clinical approaches have not yet led to effective treatments for AD, highlighting the urgent need for a better understanding of the underlying mechanisms that cause cognitive decline in this condition (Kuo and Rajesh 2019; Passeri et al. 2022; Ratan et al. 2023).

The hippocampus plays a crucial role in processing spatial memory and is significantly affected in AD patients from the early stages (Philippen et al. 2024; Billaud and Yu 2024). Initial investigations of post-mortem AD brains have revealed significant disturbances in the hippocampal structure and connectivity, including catecholaminergic projections from the Ventral Tegmental Area (VTA) and Locus Coeruleus (LC) (Sala et al. 2021; Dai et al. 2023). Additionally, AD murine models show diminished catecholaminergic innervation within the hippocampus, correlating with alterations in neuronal excitability and modified gamma-wave oscillatory activity (Nobili et al. 2017; Sakai et al. 2023; Spoleti et al. 2024). Disturbances of the catecholaminergic system, such as decreased catecholamine neurotransmitter levels (Pan et al. 2020) and quantities of catecholamine receptors (Kemppainen et al. 2003; Szot et al. 2006), are common in AD. Damage to catecholaminergic neurons has been linked to memory deficits in animal models of AD and may contribute to the initial symptoms experienced by AD patients (Gloria et al. 2021; Iannitelli et al. 2023; Bueichekú et al. 2024). Moreover, several studies have suggested the crucial role of catecholaminergic transmission in improving memory deficits in AD. Pharmacological activation of the catecholaminergic system through the use of dopamine (DA) agonists, the DA precursor L-DOPA, or the monoamine oxidase-B inhibitor selegiline has been shown to enhance synaptic plasticity, increase dendritic spine density, modulate hippocampal postsynaptic composition, and improve memory deficits in experimental AD models (Swanson-Park et al. 1999; Yuan Xiang et al. 2016; Moreno-Castilla et al. 2016; Tábi et al. 2020; Alborghetti et al. 2024; Basir et al. 2024).

Recent research has shifted the focus from the VTA to the LC as a significant catecholaminergic neuromodulatory structure within the hippocampal circuitry, affecting memory modulation. Studies have shown that triple transgenic mice modeling AD (3xTg AD) display dysfunction in the dopaminergic neurons of the VTA. These findings suggest that 3xTgAD mice exhibit deficits in reward-based learning due to dysregulated DA neurons in the VTA. This dysregulation is characterized by hyperexcitability and disrupted firing, resulting from the decreased activity of small-conductance calcium-activated potassium (SK) channels (Blankenship et al. 2024). Additionally, other studies have shown increased D2/3 receptor density in the striatum and heightened sensitivity of D2 receptors, alongside reduced anxiety-like behavior and increased locomotion in 12-month 3xTg-AD mice, highlighting the catecholaminergic fiber deficit in AD (Gloria et al. 2021). Although the reduction in VTA activity has been linked to a decline in behavioral tasks (Shan et al. 2023), whether the activity of VTA neurons reverses cognitive impairments in AD mice remains an open question. Previous studies have shown that 3xTgAD mice exhibit an age-related loss of TH + neurons in the LC, with a 26% reduction in these neurons at 12 months (Manaye et al. 2013). The LC sends denser catecholaminergic inputs to the CA1 region of the hippocampus than the VTA, and it has been found to modulate synaptic plasticity and spatial memory processes (Takeuchi et al. 2016; James et al. 2021; Gálvez-Márquez et al. 2022). In the early stages of AD, damage and degeneration of catecholaminergic neurons may occur as a direct consequence of high energy expenditure (Wimalasena et al. 2024) and significant perturbations in intracellular calcium (Ca2+) mediated by Aβ accumulation (Bezprozvanny and Mattson 2008), leading to oxidative stress. This damage may be further exacerbated by the oxidation of DA, resulting in the production of dopaquinone and reactive oxygen species. Increased oxidative stress is associated with higher expression of pro-inflammatory cytokines, which disrupt synaptic efficiency (Tönnies and Trushina 2017). Additionally, the α2A adrenergic receptors, which are highly expressed in the LC, are implicated in the development of tau tangles through the GSK3β/tau signaling cascade (Braak and Del Tredici 2004; Ferrari et al. 2006; Khaliq and Bean 2010; Surmeier et al. 2017; Zhang et al. 2018; Matchett et al. 2021; Guzmán-Ramos et al. 2022). This process may contribute to the degeneration of LC catecholaminergic neurons in both AD mouse models and human patients (Chen et al. 2013; Dahl et al. 2023). It has also been associated with decreased levels of NA and DA) in individuals with AD and in animal models, which correlates with memory decline (Kelly et al. 2017). Other studies indicate that noradrenaline (NA) levels can be increased with the NA precursor L-threo-3,4-dihydroxyphenylserine (L-DOPS) in 5-month-old 5xFAD mice. Treatment with L-DOPS has been shown to improve performance in the Morris Water Maze (MWM), a memory task dependent on the hippocampus (Kalinin et al. 2012). Additionally, chemogenetic activation of the LC has been shown to restore reversal learning in a rat model of AD (Rorabaugh et al. 2017). However, it remains unclear whether restoring catecholaminergic transmission from the LC to the hippocampus can alleviate the memory deficits associated with AD.

To study the effects of stimulating LC catecholaminergic transmission on hippocampal synaptic plasticity and spatial memory retrieval, we selectively stimulated the catecholaminergic projections from the LC into the CA1 region of the dorsal hippocampus in a modified 3xTgAD mouse model (AD-TH), which expresses the CRE recombinase protein under the control of the tyrosine hydroxylase (TH) promoter. We used optogenetic stimulation to activate the LC catecholaminergic projections into the CA1 hippocampal region before the retrieval session in two different spatial memory protocols: the Morris Water Maze (MWM) and Object Location Memory (OLM). Our findings indicate that optogenetic stimulation of the LC catecholaminergic projections into the CA1 hippocampal region before memory retrieval effectively reversed memory impairment in AD mice.

We suggest that the spatial memory retrieval deficit observed in AD-TH mice is partially caused by reduced basal levels of catecholamines in the CA1 region of the hippocampus. This reduction is linked to fewer catecholaminergic projections from the LC and impaired synaptic plasticity in the same region of the hippocampus in aged mice. Interestingly, optogenetic stimulation of these LC catecholaminergic projections restored the levels of catecholamines and synaptic plasticity in the CA1 hippocampal region in these mice. These findings suggest potential therapeutic implications for targeting LC catecholaminergic pathways in AD-related cognitive decline.

## Materials and methods

### Animals

C57BL/6J wild-type mice (WT), along with 3xTgAD and TH-Cre x 3xTgAD (AD-TH) transgenic male and female mice, aged 3 (young) and 12 months (aging), were used during the optogenetic, electrophysiological, and behavioral experiments. The AD-TH strain was created by breeding 3xTgAD mice (Guzmán-Ramos et al. 2012; Moreno-Castilla et al. 2016) with TH-Cre mice (Gálvez-Márquez et al. 2022). Genotyping was performed to confirm the presence of the presenilin 1, amyloid precursor protein (APP), human tau, and Cre genes in the offspring. These mice were selectively bred for at least four generations alongside 3xTgAD mice to establish a strain expressing presenilin 1, APP, and tau proteins, with Cre-recombinase regulated by the TH promoter. For genotyping, we used the hotshot method. A tail snip was lysed in an alkaline reagent of 25 mM NaOH and 0.2 mM disodium EDTA at 95 °C for 1 h. After lysis, the DNA was neutralized using 1 M Tris-HCl (pH 7.4) and centrifugated at 2500 rpm for 2 min in a Hermie Z 233 MK-2 centrifuge. Finally, the DNA supernatant was collected. This supernatant was amplified using PCR (specific protocol number from QIAGEN) and analyzed via agarose gel electrophoresis.

The mice were acquired from the Institute of Cellular Physiology animal facility at the National Autonomous University of Mexico. They were individually housed in transparent acrylic cages with ad libitum access to food and water, maintained under a 12/12-hour light/dark cycle at a temperature of 22 ± 2 °C and relative humidity of 50 ± 5%. Previous research has shown that learning impairment is more pronounced during the light phase (Giménez-Llort et al. 2021); therefore, all experimental procedures were conducted during this phase of the cycle, which is a well-established protocol for AD research. Ethical approval was obtained from the Institutional Animal Care and Use Committee of the Institute of Cellular Physiology (Approval No. FBR125-18), by the guidelines outlined in the Official Mexican Standard NOM-062-ZOO-1999.

### Stereotaxic surgery

All animals were initially anesthetized using a mixture of oxygen (1 L per min) and isoflurane (induction 5%; Maintenance 1–2%; Vip 3000 matrix) before being secured in a stereotaxic apparatus (RWM Life Science, Texas, USA). Surgical coordinates were referenced from the Allen Brain (Atlas Allen Institute for Brain Science). For optogenetic experiments, AD-TH mice were bilaterally injected with either Channelrhodopsin (ChR2) or an empty adeno-associated virus vector. Specifically, the ChR2 group received 0.5 µL per hemisphere of rAAV5/EfIα-DIO-hChR2 (viral concentration of 5.2 × 10^12^ virus molecules/ml), while the control group received 0.5 µL per hemisphere of rAAV5/EfIα-DIO-eYFP (with a viral concentration of 6.0 × 10^12^ virus molecules/ml, where eYFP stands for enhanced yellow fluorescent protein). The injections were targeted to the LC at coordinates − 5.5 mm anterior-posterior (AP), ± 0.9 mm medial-lateral (ML), and − 3.3 mm dorsal-ventral (DV) relative to bregma. The virus was administered using calibrated glass micropipettes (5 µL, Drummond, USA). Additionally, optical fibers were bilaterally implanted in the dorsal hippocampus CA1 at coordinates − 2.40 mm AP, ± 2.0 mm ML, and − 1.00 mm DV relative to bregma. Mice used for the microdialysis experiments were implanted with an optical fiber (0.22 NA, 200 μm diameter; Doric Lenses, Canada) into the CA1 region of the hippocampus (− 2.40 mm AP; ±2.0 mm ML; −1.00 mm DV to bregma). A cannula guide (CMA/7; CMA Microdialysis, Sweden) was also bilaterally implanted into the CA1 (− 3.0 mm AP; ±2.0 mm ML; −1.5 mm DV to bregma) at a 25º frontal angle. After surgery, mice were allowed to recover for two weeks before beginning the experiments.

### Object location memory (OLM) task

The OLM task was conducted in an open field inside a gray wooden box (33 × 33 × 30 cm) covered with a thin layer of sawdust. A spatial cue was positioned on one of the walls of the box. Two different Lego figures (5 × 5 × 5 cm) were used as objects to be explored. Using Debut Video Capture-NCH software version 5.73, a video camera was positioned above the box to record the sessions. Mice were habituated to the box for 10 min without any objects for three consecutive days. During the following two acquisition sessions (10 min each day), the mice were allowed to explore two objects placed in specific positions. After a 24-hour interval, the long-term memory (LTM) test was conducted. During this test, the mice were allowed to explore the two objects for 10 min in freely movement; however, one of the objects was relocated to a novel position for the trial. The novel and familiar positions of the objects were always counterbalanced to prevent any side preference in the arena. The boxes and objects were deodorized with 70% ethanol, and the sawdust bedding was changed between trials. The AD-TH mouse groups (eYFP and ChR2) received optogenetic stimulation (473 nm laser, 10–15 mW, 20 Hz, 10 min at 5 ms pulses; 1 s light-on, 4 s light-off at 15 mW; OEM Laser Systems, USA), this stimulation is based on previous reports with LC stimulation (Takeuchi et al. 2016; Kempadoo et al. 2016) in the hippocampal CA1 region during the LTM test. The sessions were recorded and analyzed offline, focusing on the total exploration time for each object during the acquisition and LTM sessions. The recognition index was calculated by dividing the exploration time for each object by the total exploration time for both objects during each session. It was expected that the mice’s recognition index would be close to 0.5 during the acquisition phase, indicating no preference for any object, to be included in the statistical analysis. Our exclusion criteria stated that a mouse with a recognition index greater than 0.8 or less than 100 centi-seconds of total exploration would be excluded from the analysis.

### Morris water maze (MWM) task

A MWM behavioral task was conducted in a circular pool (110 cm in diameter) with a white bottom. An escape platform (15 cm x 15 cm x 15 cm) was positioned at a fixed location 0.5 cm under the water level to remain invisible to the mice. The water was opaque, and a special non-toxic white paint was used. Two geometric figures placed in opposite positions on the pool walls were used as spatial cues. A video camera was placed above the pool to record the trials during the acquisition and the LTM sessions using Debut Video Capture-NCH software version 5.73. Mice were handled for three minutes three days before the acquisition sessions to reduce stress. The acquisition sessions consisted of four training trials daily for four days. Animals were placed at different pre-established starting positions for each trial, with the experimenter always positioned in the same place, serving as a spatial cue. Mice were allowed to swim for 60 s and reach the escape platform. The time to reach the platform was recorded; if the mice could not find the escape platform within 60 s, the experimenter guided them. Once on the platform, the mice were allowed to explore for 30 s. After each trial, each mouse was placed in an open box for 60 s to rest before returning to the pool for the subsequent trial. Our exclusion criteria were if the mouse had an average latency time to the platform of more than 30 s in the last training trial.

The LTM test was conducted 48 h after the last training session. During the LTM test, the escape platform was removed from the pool, and mice were allowed to swim freely for 60 s. AD-TH mouse groups (eYFP and ChR2) were optogenetically stimulated (473 nm laser, 10–15 mW, 20 Hz, 5 min at 5 ms pulses; 1 s light-on, 4 s light-off at 15 mW; OEM Laser Systems, USA) in the hippocampal CA1 immediately before the LTM test (Kempadoo et al. 2016). Video records were analyzed offline to determine the number of platform crossings, time in the target quadrant, and swimming speed. The video was utilized to determine the area and location of the escape platform during the LTM sessions. The time each mouse spent reaching this area (latency to platform) and the number of times mice crossed to the area (number of crosses) were quantified. For the time analysis in the target quadrant, the pool was divided into four equal quadrants in the video, and the quadrant containing the escape platform was defined as the target quadrant. The swimming time of mice in the target quadrant was quantified. Swimming speed was quantified using the Image J tracking function.

### Electrophysiology

The mice were anesthetized with sodium pentobarbital, initially at a dose of 20 mg/kg of body weight, and then a maintenance dose of 10 mg/kg was administered 60 min later. Once anesthetized, the mice were positioned in a stereotaxic apparatus (51603, Stoelting, Chicago, USA). A skin incision was made to expose the skull, and a stainless-steel concentric bipolar stimulation electrode was implanted in the CA3 region of the hippocampus (coordinates from bregma: AP 1.5 mm, ML 2.0 mm, DV 1.2 mm). A stainless-steel monopolar electrode was placed in the CA1 region of the hippocampus (coordinates from bregma: AP −2.2 mm, ML 1.4 mm, DV 1.0 mm) to record responses. Stimulation was provided using an AM Systems Model 2100 stimulator and delivered to the stimulation electrode. After establishing a baseline electrically evoked response for 15 min with a stimulation intensity set at 50% of the maximum EPSP amplitude, a long-term potentiation (LTP) protocol was initiated. This involved delivering four stimulation trains (1 s at 100 Hz) with 20-second intervals between each train, referred to as high-frequency stimulation (HFS). The evoked responses following this stimulation were recorded for an hour, and field excitatory post-synaptic potentials (fEPSP) were calculated as the percentage change in evoked response amplitude compared to the baseline. AD-TH mouse groups (eYFP and ChR2) were optogenetically stimulated using (473 nm laser, 10–15 mW, 20 Hz, 15 min at 5 ms pulses; 1 s light-on, 4 s light-off at 15 mW; OEM Laser Systems, USA) for 5 min before the HFS and 10 min after the HFS. Offline analysis was conducted using the fEPSP recorded before and after the HFS.

### Microdialysis

A CMA/7 membrane (CMA Microdialysis, Sweden) was inserted into the guide cannula previously implanted in mice. Ringer solution (MgCl2 12mM, NaCl 1.44 M, CaCl2 17 mM and KCl 48 mM) was perfused at a 0.25 µl/min rate using a micro-infusion continuous pump (100 pump CMA Microdialysis, Sweden). Mice were placed into the arena without objects and infused with Ringer solution to collect the neurotransmitter using the EICOM piping system (Concise Freely Moving System, EICOM, USA). Once the 30-minute stabilization period concluded, three 16-minute samples were collected to calculate the baseline. These samples were placed into a vial containing an antioxidant blend (Ascorbic acid 25 mM, Na2EDTA 27 mM, and acetic acid 1 M). Subsequently, a fourth fraction was collected while the mice (eYFP and ChR2) were optogenetically stimulated (473 nm laser, 10–15 mW, 20 Hz, 15 min at 5 ms pulses; 1 s light-on, 4 s light-off at 15 mW; OEM Laser Systems, USA), and two post-stimulation samples were also collected. All samples were stored at −80 °C.

### Neurotransmitter analysis

Neurotransmitter concentration was quantified by capillary electrophoresis. Briefly, all microdialysis samples were prepared for derivatization by adding 6µL of 3-(2-Furoyl)-quinoline-2-carboxaldehyde (FQ, 16.67 mM, Molecular Probes; Massachusetts Invitrogen, USA) and catalyzed with 2 µL KCN (24.5 mM) in borate buffer (10 mM pH 9.2). 1 µL of an internal standard (0.075 mM, O-methyl-L-threonine; Fluka, Indiana, USA) was added to each microdialysis sample, and they were incubated in the dark for 15 min at 65 °C. Neurotransmitters were quantified with laser-induced fluorescence (LIF) detection within a capillary electrophoresis system (P/ACE MDQ, Beckman Coulter; Pasadena, USA). Compound separation was based on the micelle electrokinetic chromatography method. Microdialysis samples were hydrodynamically injected into the capillary system at 0.5 psi for 5s. Separation occurred in a buffer (borates 35 mM, sodium dodecyl sulfate 25 mM, and 13% methanol HPLC grade, pH 9.6), at neurotransmitters were detected by fluorescence using a LIF device; light at 488 nm from an argon ion laser was used to excite the FQ-labeled analytes. Signals were depicted as electropherograms and analyzed offline using 32Karat TM8.0 software (Beckman Coulter, Pasadena, USA). Neurotransmitters were identified by comparison with the standard electropherogram pattern (DA and NA standards). Signals were quantified by measuring the ratio of the area under the curve for each neurotransmitter and the area under the curve of its respective internal standard.

### Immunofluorescence and confocal microscopy

Mice were euthanized utilizing sodium pentobarbital (75 mg/kg) after the behavioral, electrophysiological, and neurotransmitter analysis experiments. Immediately after euthanasia, mice were perfused with a 0.9% saline solution and fixed with a 4% paraformaldehyde solution. The brain was extracted and preserved in a 4% paraformaldehyde solution. A cryostat (Leica CM520, Germany) cut mice brains in 40 μm coronal slices. Free-floating tissue slices were incubated overnight with primary antibodies diluted in a 5% bovine serum albumin buffer (NaCl 150 mM, Triton X-100 0.1%, Trizma base 100 mM, pH 7.4) at a dilution of 1:1000 (mouse monoclonal anti-human phospho-PHF-tau pSer202/Thr205 antibody, Thermo Scientific, Belgium; mouse monoclonal BAM-10 anti-beta-amyloid antibody, Missouri, Sigma Aldrich, USA; rabbit polyclonal anti-TH antibody, Arkansas, Pel-Freez, USA). In the case of immunohistochemistry against Aβ, the floating tissues were incubated for 5 min in formic acid and washed with TBS-T 0.1% before the primary antibody incubation. The next day, floating slices were washed 6 times with Tris-buffered saline solution and then incubated with secondary antibodies diluted in 5% bovine serum albumin buffer at a 1:500 dilution (Mouse IgG antibody (FITC), California, GeneTex, USA; Gt X Rb IgG Cy3, Massachusetts, Millipore, USA) for 2 h. After that, the slices were mounted using a Dako fluorescence mounting medium. The immunofluorescence was examined using a ZEISS LSM 800 confocal microscope (Zeiss, Germany). The immunofluorescence images were digitized for further analysis using Image J (version), and the antibody signal was obtained and quantified by measuring the percentage of the pixel area.

### Statistics

Statistical analysis was performed using GraphPad Prism software (version 7.00, USA). All graphs presented mean ± standard error of the mean (SEM), with statistical significance defined as *p* < 0.05. For immunohistochemistry, images were processed using ImageJ software and analyzed with one-way ANOVA followed by Fisher’s LSD post hoc test. Data from the OLM and MWM tasks were analyzed using one-factor or two-factor ANOVA, followed by multiple comparison tests with significance determined using Fisher’s LSD. Microdialysis analyses were conducted using one-way ANOVA with Fisher’s LSD post hoc analysis. Electrophysiological data were also analyzed using one-way ANOVA followed by Fisher’s LSD post hoc analysis.

## Results

### Optogenetic stimulation of the LC-CA1 projections enhances spatial memory retrieval in an aging AD-TH mouse model

**Fig. 1:**
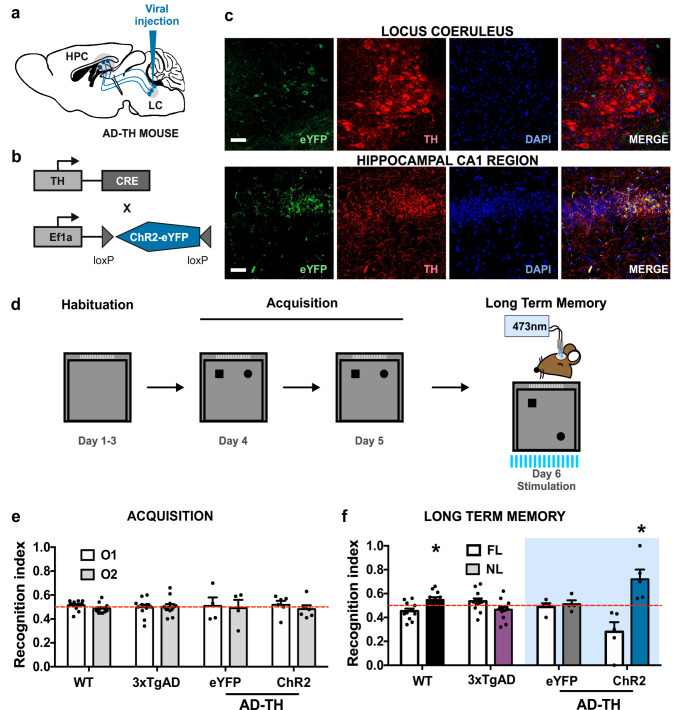
Optogenetic stimulation of catecholaminergic LC-CA1 projection improves memory retrieval in aging AD-TH mice in OLM protocol.** a** Schematic representation of the bilateral adeno-associated virus vector injection of AAV-ChR2 or AAV-eYFP within the LC in AD-TH mice. **b** The adeno-associated virus vector of AAV-ChR2 and a diagram of the CRE recombinase protein under the control of the TH promoter are depicted. **c** Representative images of coronal sections of LC and hippocampal CA1 projection from LC are shown. eYFP (green), TH (red), DAPI (blue), and MERGE (colocalization). **d** Schematic diagram of the OLM protocol. Optogenetic stimulation of the catecholaminergic LC projections within the CA1 hippocampal region was delivered along the 10-minute LTM session (20 Hz, 5 ms, 473 nm to 10–15 mW). **e** Average of the recognition index during the acquisition sessions for the WT (*n* = 11, 12 months old), 3xTgAD (*n* = 11, 12 months old), and AD-TH eYFP (*n* = 4, 12 months old) AD-TH ChR2 (*n* = 5, 12 months old) mice groups. No preference to explore either of the objects is observed in any of the mice groups (Acquisition; Two-way-ANOVA: groups factor, F (Basir et al. 2024; Maity et al. 2022) < 0.0001, *P* > 0.9999; between objects, F(Abramov et al. 2009; Maity et al. 2022) = 0.9169, *P* = 0.3423). **f** During the LTM, the location of one object changed (NL) (LTM; Two-way-ANOVA: groups factor, F (Basir et al. 2024; Lyness et al. 2003) < 0.0001, *P* > 0.9999; between objects, F (Abramov et al. 2009; Lyness et al. 2003) = 19.65, *P* < 0.0001). WT mice (average exploration time: 7.90 ± 1.02 s) recognized the object in novel location vs. familiar location (Fisher’s LSD, t(Lyness et al. 2003) = 2.243, *P* = 0.0289) with a statistical power (1- β err prob) = 0.9146., however 3xTgAD (average exploration time: 5.62 ± 1.16 s) and AD-TH eYFP mice (average exploration time: 7.17 ± 1.63 s) failed to recognized the object in novel location vs. familiar location (Fisher’s LSD, 3xTgAD: t(Lyness et al. 2003) = 1.735, *P* = 0.0883, AD-TH eYFP: t(Lyness et al. 2003) = 0.3577, *P* = 0.7219). Finally, AD-TH ChR2 mice (average exploration time: 2.78 ± 7.9 s) recognized the object in a novel location vs. a familiar location (Fisher’s LSD, t (Lyness et al. 2003) = 7.038, *P* < 0.0001) with a statistical power (1- β err prob) = 0.9396. The analysis of NL recognition between the WT and ChR2 showed a statistical difference (Fisher’s LSD, NL-WT vs. NL-ChR2: t(Lyness et al. 2003) = 3.24, *P* = 0.002). The red dotted horizontal line represents the 0.5 recognition index threshold. The blue bars indicate the optogenetic stimulation. All results showed the mean ± SEM. *P < 0.05. HPC: Hippocampus; s: seconds. Scale bar: 50 μm

Research has demonstrated the significance of catecholamines in spatial memory, particularly using optogenetic techniques. Studies have shown that inhibiting catecholaminergic projections from the LC to the hippocampal CA1 region results in impaired spatial memory expression (Gálvez-Márquez et al. 2022). Notably, these projections have been reduced in AD mice (Theofilas et al. 2017; James et al. 2021). This finding motivated us to investigate the effects of optogenetic activation of LC projections on hippocampal-dependent memory and synaptic plasticity in 12-month-old 3xTgAD mice. We used an adeno-associated virus vector to express the channelrhodopsin-2 (ChR2) protein or the enhanced yellow fluorescent protein (eYFP) reporter protein in LC neurons. Additionally, we implanted an optic fiber into the CA1 region of the hippocampus of our modified 12-month-old AD-TH mouse model (see Fig. 1a and b). To evaluate the efficacy of viral infection in the LC neurons of the AD-TH mice and their projections to the hippocampus (Fig. 1a and b), we conducted an immunohistochemical analysis of the eYFP reporter protein. This analysis assessed its expression in LC tyrosine hydroxylase-positive (TH+) neurons and their projections within the CA1 hippocampal region. As shown in Fig. 1c, the eYFP reporter protein is expressed in the LC neurons that project to the CA1 region of the hippocampus. Furthermore, the TH immunoreactive signal colocalizes with the eYFP immunoreactive signal in both brain regions (Fig. 1c). These results indicate similar expression of the adeno-associated virus vector in the LC and its projections in the hippocampus, aligning with findings from previous studies (Takeuchi et al. 2016; Kempadoo et al. 2016; Gálvez-Márquez et al. 2022).

We implemented a behavioral paradigm to assess hippocampal-dependent OLM (Fig. 1d) and evaluate the effects of optogenetic stimulation of catecholaminergic LC projections within the CA1 region. During the two-day acquisition phase, all aging mouse groups exhibited comparable exploration of both objects, as reflected by similar recognition indices, indicating no preference for either object (Fig. 1e). Twenty-four hours later, during a 10-minute LTM test, aging AD-TH mice received optogenetic stimulation. While aging WT mice successfully recognized the object in the novel location, both aging 3xTgAD and AD-TH eYFP control mice failed to distinguish between the novel and familiar locations, suggesting impaired LTM retrieval. In contrast, optogenetic stimulation of LC-CA1 projections in AD-TH ChR2 mice significantly improved OLM retrieval, as demonstrated by a higher recognition index for the object in the novel location (Fig. 1f). Additionally, the optogenetic stimulation increased the recognition index of NL in the AD-TH ChR2 compared with NL in the WT.

**Fig. 2:**
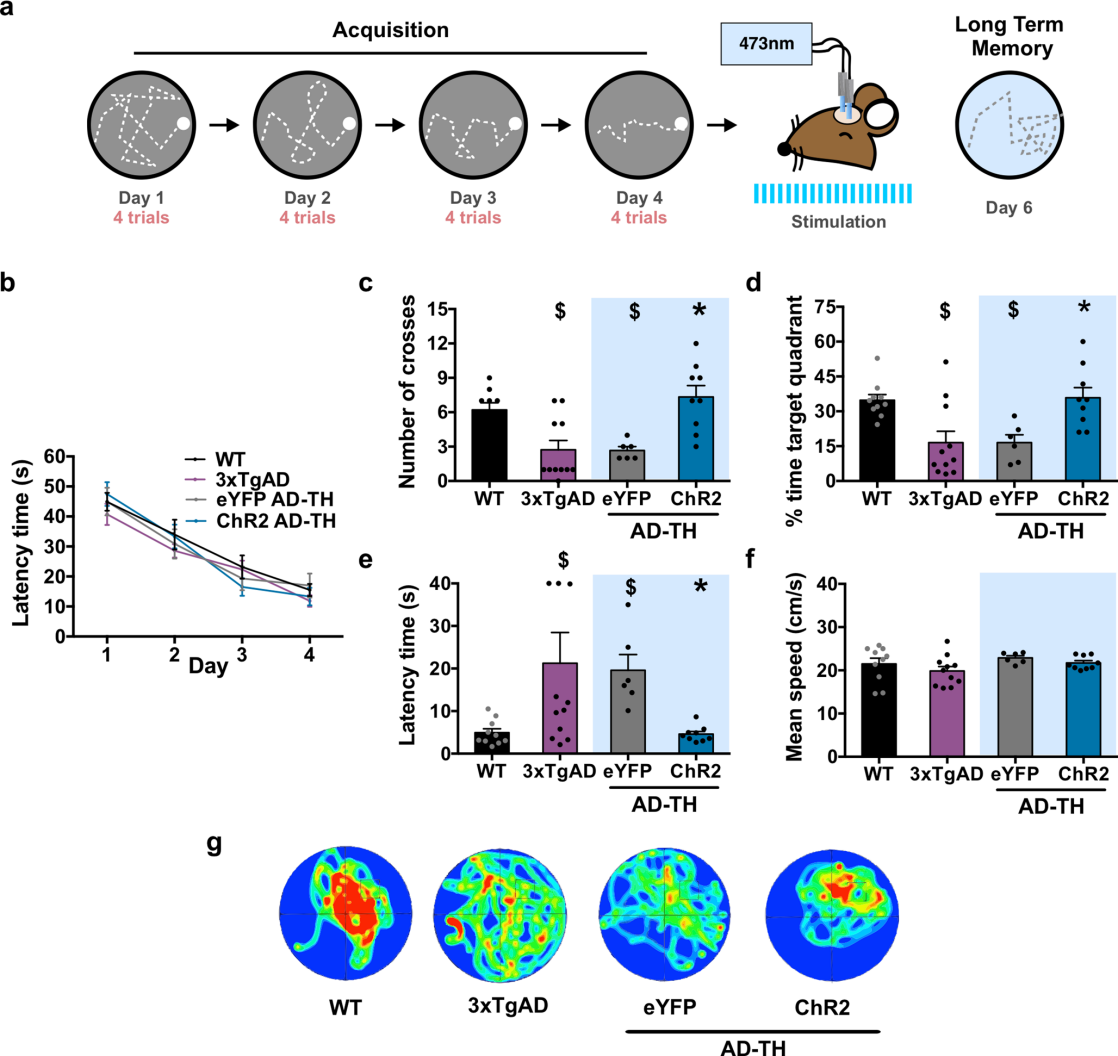
Optogenetic stimulation of catecholaminergic LC-CA1 projection reverses the retrieval deficits in the MWM spatial memory in AD-TH mice in 12 months old.** a** Schematic diagram of the MWM protocol. Optogenetic stimulation was delivered 5 min before the LTM test for the AD-TH ChR2 and AD-TH eYFP mice groups. **b** The latency time to reach the hidden platform was recorded during a four-day acquisition session for the 12 month old mouse groups: wild-type (WT, *n* = 10), 3xTgAD (*n* = 11), AD-TH ChR2 (*n* = 9), and AD-TH eYFP (*n* = 6). All groups of mice showed improved performance in reaching the hidden platform over time, with no significant statistical differences observed between groups (Repeated measures two-way ANOVA: groups F(Basir et al. 2024; Gutiérrez et al. 2022) = 0.4566, *P* = 0.7146; time F(Basir et al. 2024; Titulaer et al. 2021) = 55.79, *P* < 0.0001). The following Fisher’s LSD comparisons were also significant: WT latency time from day 1 to day 4: t(Titulaer et al. 2021) = 6.377, *P* < 0.0001; 3xTgAD latency time from day 1 to day 4: t(Titulaer et al. 2021) = 5.239, *P* < 0.0001; AD-TH eYFP latency time from day 1 to day 4: t(Titulaer et al. 2021) = 5.085, *P* < 0.0001; AD-TH ChR2 latency time from day 1 to day 4: t(Titulaer et al. 2021) = 7.774, *P* < 0.0001. Forty-eight hours later, an LTM session was conducted, and several parameters were measured. **c** The number of crossings to the area of the platform (one-way ANOVA results showed a significant effect (F(Basir et al. 2024; Guzmán-Ramos et al. 2022) = 8.945, *P* = 0.0002). The 3xTgAD and AD-TH eYFP mice demonstrated a significantly lower crossings to the platform area than WT mice. In contrast, the AD-TH ChR2 mice had several crossings similar to those of WT mice. The detailed comparisons were as follows: WT vs. 3xTgAD: t(Guzmán-Ramos et al. 2012) = 3.341, *P* = 0.0021; WT vs. AD-TH eYFP: t(Guzmán-Ramos et al. 2012) = 2.876, *P* = 0.0071; WT vs. AD-TH ChR2: t(Guzmán-Ramos et al. 2022) = 0.6390, *P* = 0.7775; AD-TH ChR2 vs. AD-TH eYFP: t(Guzmán-Ramos et al. 2022) = 3.3270, *P* = 0.0102. **d** Percent time in the target quadrant was analyzed using a one-way ANOVA, revealing a significant effect (F(Basir et al. 2024; Guzmán-Ramos et al. 2012) = 6.912, *P* = 0.001). Both the 3xTgAD and AD-TH eYFP groups spent less time in the target quadrant than the wild-type (WT) group. Additionally, the AD-TH ChR2 group exhibited a percentage of time in the quadrant comparable to that of the WT group, performing better than the AD-TH eYFP group. Subsequent analysis using Fisher’s LSD showed the following results: WT vs. 3xTgAD (t(Guzmán-Ramos et al. 2012) = 3.383, *P* = 0.0019); WT vs. AD-TH eYFP (t(Guzmán-Ramos et al. 2012) = 2.862, *P* = 0.0074); WT vs. AD-TH ChR2 (t(Guzmán-Ramos et al. 2012) = 0.1874, *P* = 0.8525); AD-TH ChR2 vs. AD-TH eYFP (t(Guzmán-Ramos et al. 2012) = 2.968, *P* = 0.0056). **e** The latency time to reach the platform area was also measured, showing significant results (one-way ANOVA, F(Basir et al. 2024; Guzmán-Ramos et al. 2012) = 3.928, *P* = 0.0171). The 3xTgAD and AD-TH eYFP groups displayed longer latency times compared to the WT group (Fisher’s LSD results: WT vs. 3xTgAD, t(Guzmán-Ramos et al. 2012) = 2.686, *P* = 0.0114; WT vs. AD-TH eYFP, t(Guzmán-Ramos et al. 2012) = 2.043, *P* = 0.0494). In contrast, the AD-TH groups showed improved LTM performance, with shorter latency times than the WT group. Specifically, the AD-TH ChR2 group performed better than the AD-TH eYFP group (Fisher’s LSD: WT vs. AD-TH ChR2, t(Guzmán-Ramos et al. 2012) = 0.0525, *P* = 0.9584; AD-TH ChR2 vs. AD-TH eYFP, t(Guzmán-Ramos et al. 2012) = 2.048, *P* = 0.0489). **f** Swimming speed was also measured to ensure that any motor deficits would not influence the results. No statistically significant differences were noted between the mouse groups regarding mean speed (one-way ANOVA, F(Basir et al. 2024; Guzmán-Ramos et al. 2012) = 1.425, *P* = 0.2537). **g** Representative heat maps illustrated the average trajectory of the mice in each group; blue indicates less time spent exploring, while red indicates more time spent exploring. The blue bar indicates periods of optogenetic stimulation. All results are presented as mean ± SEM. Notation: $ indicates *P* < 0.05 compared with WT; * indicates *P* < 0.05 compared with eYFP. HPC: Hippocampus

To further validate these results, we employed the MWM paradigm to assess the impact of LC-CA1 optogenetic activation on spatial memory retrieval. Mice underwent four training trials each day over four days during the acquisition phase (Fig. 2a). No significant differences in latency time to reach the hidden platform were observed among the aging WT, 3xTgAD, and AD-TH mice groups during training. By the fourth day, all groups showed reduced latency time to reach the hidden platform (Fig. 2b), indicating intact spatial learning. However, during the LTM test, aging AD mice displayed deficits in spatial memory retrieval compared to aging WT mice. Both 3xTgAD and AD-TH eYFP mice showed fewer platform crossings (Fig. 2c), spent less time in the target quadrant (Fig. 2d), and exhibited longer latencies to reach the platform area (Fig. 2e). These findings confirm impaired spatial memory retrieval in the AD mouse models tested, similar to OLM.

Interestingly, aging AD-TH ChR2 mice showed improved spatial memory retrieval when a five-minute optogenetic stimulation of catecholaminergic LC-CA1 projections was administered immediately before the LTM test. This improvement was evidenced by an increased number of platform crossings, a greater percentage of time spent in the target quadrant, and a reduced latency to reach the platform area compared to aging AD-TH eYFP mice, reaching levels comparable to those of aging WT mice (Fig. 2c, d, and e). Importantly, swimming speed remained consistent across all groups, indicating that the observed improvements were not due to motor changes induced by the optogenetic stimulation (Fig. 2f), and we observed a similar swimming course between WT and AD-TH ChR2 (Fig. 2g). These findings support the hypothesis that activation of catecholaminergic LC projections within the CA1 region enhances spatial memory retrieval, as assessed by both the OLM and MWM paradigms.

Finally, to demonstrate that AD-related symptoms are present in aging but not in young mice and to confirm that the genetic background of AD-TH mice does not interfere with the typical development of memory deficits, we evaluated the performance of young WT, 3xTgAD, and AD-TH mice using both the OLM and MWM paradigms. Young 3xTgAD and AD-TH mice did not exhibit impairments in LTM retrieval in OLM, as evidenced by a preference to explore the object in the novel location over the familiar one (Supplementary Fig. 1a, b, and c). Total exploration time was also comparable across groups (Supplementary Fig. 1 d), indicating that motivation and exploratory behavior were unaffected in young mice. On the other hand, during the LTM test in the MWM, all groups of young mice, including WT, AD-TH, and 3xTgAD mice, showed normal spatial memory retrieval (Supplementary Fig. 2b, c, and e) and no differences in speed among the groups (Supplementary Fig. 2f).

Overall, these results suggest that although memory acquisition remains intact in both young and aged AD mice, age-dependent deficits in memory retrieval emerge under pathological conditions in aged AD mice. Furthermore, targeted activation of the LC to CA1 projections can effectively alleviate the retrieval deficits observed in aged AD mice.

### Optogenetic stimulation of the LC-CA1 projections enables LTP induction in aging AD mice

**Fig. 3:**
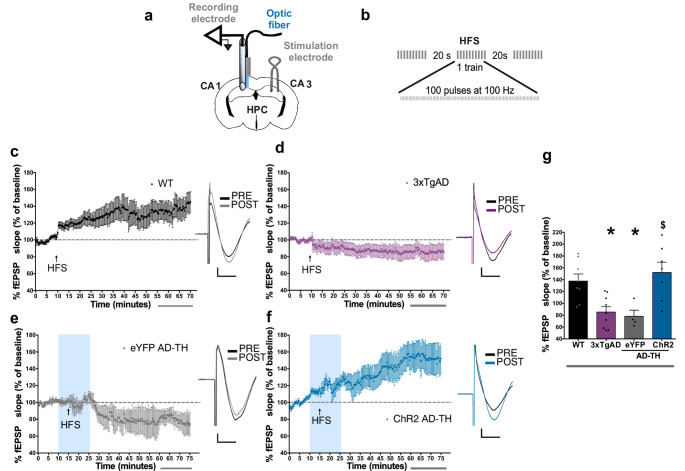
Optogenetic stimulation of the catecholaminergic LC-CA1 projection restores the synaptic plasticity in aging AD-TH mice.** a** The diagram depicts the optic fiber, recording electrode, and stimulation electrode in the Schaffer collaterals in the CA1 hippocampus. **b** The HFS protocol involved three trains of 100 pulses at 100 Hz. **c** fEPSP recordings in WT (*n* = 8, 12 months old) mice before and after HFS with representative traces of EPSP before (PRE) and after HFS (POST) (horizontal bar: 5 ms, vertical bar: 200 mV). **d** fEPSP recordings in 3xTgAD (*n* = 9, 12 months old) mice before and after HFS with representative traces of EPSP before (PRE) and after HFS (POST). **e** fEPSP recordings in eYFP AD-TH (*n* = 4, 12 months old) mice before and after HFS with representative traces of EPSP before (PRE) and after HFS (POST). **f** fEPSP recordings in ChR2 AD-TH (*n* = 9, 12 months old) mice before and after HFS with representative traces of EPSP before (PRE) and after HFS (POST). **g** The bar graph displays the mean fEPSP recordings taken during the last fifteen minutes of measurements. A one-way ANOVA yielded a statistic of F(Basir et al. 2024; Gelinas et al. 2008) = 7.673, with a significance level of *P* < 0.0009. The results from Fisher’s LSD post-hoc tests are as follows: WT (wild type) vs. 3xTgAD: t(Gelinas et al. 2008) = 3.132, *P* = 0.0045; WT vs. AD-TH eYFP: t(Gelinas et al. 2008) = 2.838, *P* = 0.0091; WT vs. AD-TH ChR2: t(Gelinas et al. 2008) = 0.819, *P* = 0.4209; AD-TH eYFP vs. AD-TH ChR2: t(Gelinas et al. 2008) = 3.449, *P* = 0.0021. In the graph, the blue bar represents the period of optogenetic stimulation (20 Hz, 5 ms, using a 473 nm light source at 10–15 mW), and the arrow indicates HFS. All results are presented as the mean percentage of the fEPSP slope from baseline, ± SEM (standard error of the mean). Asterisks (*) denote *P* < 0.05 when compared to the WT group, while dollar signs ($) indicate *P* < 0.05 when compared to the AD-TH eYFP group

Reports have shown alterations in hippocampal synaptic plasticity in AD mice (Oddo et al. 2003; Tönnies and Trushina 2017; Brandwein and Nguyen 2019). To assess the effect of the optogenetic stimulation of LC catecholaminergic neurons within the hippocampus on the synaptic plasticity in AD mice, aging AD-TH mice were injected with the adeno-associated virus vector carrying ChR2 or eYFP reporter proteins in the LC. Aging AD-TH mice were subjected to LTP protocol coupled with the optogenetic stimulation of the catecholaminergic LC projections in the Shaffer collateral pathway of AD mice under anesthesia (Fig. 3a). LTP was induced by applying a HFS (3 trains of 100 pulses at 100 Hz) (Fig. 3b) in the CA3 projections. At the same time, the fEPSP were measured within CA1 of the hippocampus. Aging WT mice exhibited an augmented fEPSP after HFS compared to the baseline recordings (137.5% ± 12.07%), and these responses persisted for up to one hour (Fig. 3c). In contrast, aging 3xTgAD mice failed to induce LTP; instead, a depression in the fEPSP recordings following HFS was observed (85.41% ± 9.05%) (Fig. 3d). Similarly, aging eYFP AD-TH mice presented a decremented synaptic communication post-HFS (78.01.7% ± 10.35%) (Fig. 3e). However, the optogenetic stimulation of the catecholaminergic LC projections within the CA1 hippocampal region of aging AD-TH ChR2 mice reinstated the synaptic plasticity within the Schaffer collaterals pathway (152.7% ± 17.47%) after HFS (Fig. 3f). This observed potentiation of the fEPSP in aging ChR2 AD-TH mice was similar to that in aging WT mice (Fig. 3g). Interestingly, young 3xTgAD and AD-TH mice could induce and maintain a strong LTP (Supplementary Fig. 3), suggesting that alterations in the hippocampal synaptic plasticity in AD mice was not a consequence of the genetic background of the AD-TH mice, but an expression of the AD pathology.

These results indicate that enhancing the catecholaminergic LC neurotransmission within the CA1 hippocampus significantly improves the synaptic plasticity in that specific brain region. Interestingly, this reinstatement of the synaptic plasticity in the hippocampus is associated with the improved ability of AD mice to retrieve spatial memory effectively.

### Optogenetic stimulation of the LC-CA1 projections increases DA and NA levels in AD-TH mice

**Fig. 4:**
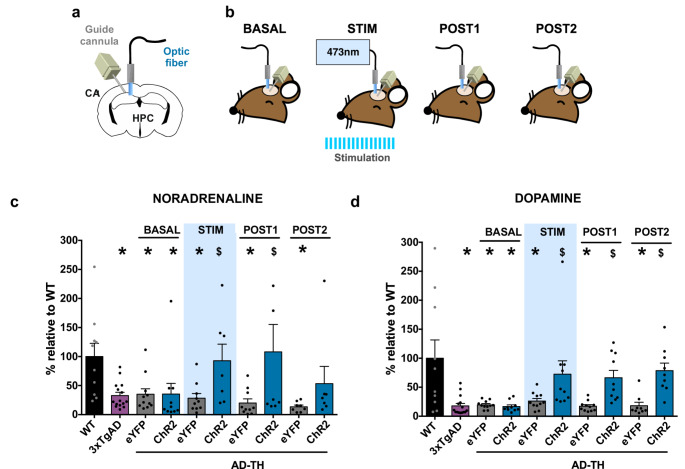
Optogenetic stimulation of the catecholaminergic LC-CA1 projection restores the catecholaminergic levels in aging AD-TH mice.** a** The diagram illustrates the implantation of the guide cannula and optic fiber in the CA1 region of the hippocampus. **b** The neurotransmitter sample collection protocol consists of three phases: before stimulation (BASAL fraction), during stimulation (STIM fraction, involving optogenetic stimulation at 20 Hz, 5 ms, 473 nm with an intensity of 10–15 mW), and after stimulation (POST1 and POST2 fractions) of the LC catecholaminergic projections into the CA1 hippocampus. **c** The percent change in norepinephrine (NA) levels was assessed relative to aging wild-type (WT) mice levels. The results revealed that the basal levels of NA were reduced in aging AD models compared to aging WT mice (*n* = 10, 12 months old). Specifically, the following were observed: 3xTGAD mice (*n* = 15, 12 months old) showed a change of 32.83% ± 5.76%, AD-TH eYFP mice (*n* = 10, 12 months old) had a change of 35.08% ± 9.40%, and AD-TH ChR2 mice (*n* = 8, 12 months old) showed a change of 35.34% ± 18.46%. Statistical one-way ANOVA analysis yielded F(Blankenship et al. 2024; Surmeier et al. 2017) = 3.105, *P* = 0.0028. Further comparisons showed significant differences: WT vs. 3xTGAD (t(Surmeier et al. 2017) = 2.778, *P* = 0.0067), WT vs. AD-TH eYFP (t(Surmeier et al. 2017) = 2.508, *P* = 0.0140), and WT vs. AD-TH ChR2 (t(Surmeier et al. 2017) = 2.441, *P* = 0.0167). Notably, optogenetic stimulation of the LC catecholaminergic projections into the CA1 hippocampus resulted in increased NA levels. Comparisons between aging AD-TH eYFP and aging AD-TH ChR2 indicated a significant difference (t(Surmeier et al. 2017) = 2.778, *P* = 0.0299). After the optogenetic stimulation, NA levels remained elevated, with POST1 measurements showing the highest levels: aging AD-TH ChR2 at 108.0% ± 47.29% and aging AD-TH eYFP at 20.04% ± 7.07%. Statistical comparisons confirmed the significance of these results (aging AD-TH eYFP vs. aging AD-TH ChR2, t(Surmeier et al. 2017) = 3.129, *P* = 0.0024). **d** The percent change in DA levels relative to aging WT mouse levels is presented here. Basal DA levels are significantly reduced compared to those observed in aging WT mice. The study involved WT mice (*n* = 10, 12 months old), 3xTgAD mice (*n* = 15, 12 months old), AD-TH eYFP mice (*n* = 11, 12 months old), and AD-TH ChR2 mice (*n* = 10, 12 months old). Analysis revealed that basal levels of NA in aging AD were lower, with aging 3xTgAD showing 17.81% ± 4.225%, aging AD-TH eYFP 19.66% ± 2.11%, and aging AD-TH ChR2 16.74% ± 2.83%. Statistical comparisons using Fisher’s LSD test showed significant differences: aging WT vs. aging 3xTgAD (t(Tse et al. 2023) = 4.740, *P* < 0.0001), aging WT vs. aging AD-TH eYFP (t(Tse et al. 2023) = 4.329, *P* < 0.0001), and aging WT vs. aging AD-TH ChR2 (t(Tse et al. 2023) = 4.383, *P* < 0.0001). Optogenetic stimulation of the LC catecholaminergic projections into the CA1 region of the hippocampus resulted in increased DA levels during stimulation. The comparison of AD-TH eYFP and AD-TH ChR2 during this stimulation was also significant (Fisher’s LSD, t(Tse et al. 2023) = 2.492, *P* = 0.0144). After the optogenetic stimulation, DA levels remained significantly elevated: POST 1 comparison showed aging AD-TH eYFP vs. aging AD-TH ChR2 (Fisher’s LSD, t(Tse et al. 2023) = 2.649, *P* = 0.0094) and POST 2 comparison (Fisher’s LSD, t(Tse et al. 2023) = 3.020, *P* = 0.0032). The blue bar in the data represents the duration of optogenetic stimulation. All results are expressed as the mean percentage change relative to aging WT mice ± standard error of the mean (SEM). Asterisks (*) indicate statistically significant differences compared to aging WT (*p* < 0.05), while dollar signs ($) indicate statistically significant differences within the aging eYFP AD-TH mice (*p* < 0.05)

Several researches have shown that the levels of catecholaminergic neurotransmitters in the hippocampus are reduced in patients with AD and corresponding mouse models (Kelly et al. 2017; Theofilas et al. 2017; Dahl et al. 2023). Based on these findings, we hypothesize that optogenetic stimulation of the TH + terminals from LC to the CA1 pathway will restore physiological levels of DA and NA in AD-TH mice. To test this hypothesis, we conducted capillary electrophoresis analysis using an in vivo microdialysis approach to quantify catecholaminergic neurotransmitter levels. Adeno-associated virus vectors encoding the channelrhodopsin-2 (ChR2) protein or the enhanced yellow fluorescent protein (eYFP) reporter protein were injected into the LC of AD-TH mice. Concurrently, neurotransmitter samples were collected from the CA1 region of the hippocampus during the optogenetic stimulation process (Fig. 4a).

Neurotransmitter samples were collected at three different time points: before stimulation (BASAL), during stimulation (STIM), and after stimulation at two intervals (POST1 and POST2) (see Fig. 4b). The analysis of NA and DA levels revealed a significant reduction in the CA1 hippocampus of aging 3xTgAD and AD-TH mice compared to aging WT mice. Optogenetic stimulation of the LC terminals in the CA1 hippocampus successfully restored extracellular levels of NA and DA for up to 30 min following stimulation (see Fig. 4c and d). These findings indicate that catecholamine concentrations in the hippocampus are reduced in aging AD mice. This decline in catecholaminergic neurotransmission, along with other pathological processes associated with AD, may contribute to the deficits in spatial memory retrieval observed in aging AD mice. Importantly, restoring catecholamine levels from the LC to the CA1 provides a biological basis for enhancing spatial memory retrieval in AD mice.

### Phenotypical characterization of the AD-TH mice

**Fig. 5:**
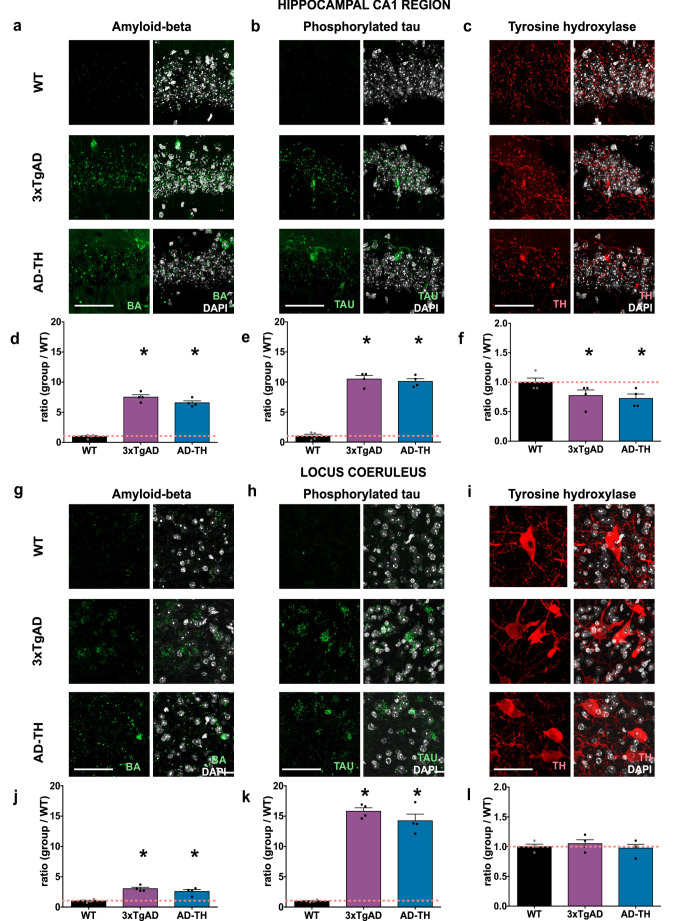
Phenotypical characterization of the aging AD-TH mice. **a** Confocal image of CA1 hippocampal coronal sections showing the immunoreactive signal for a Aβ protein (green). **b** Hyperphosphorylated tau protein (green), **c** TH (red), and DAPI (grey) in aging WT (*n* = 4, 12 months old), 3xTgAD (*n* = 4, 12 months old), and AD-TH (*n* = 4, 12 months old) mice. The ratio of the immunoreactive signal adjusted to the aging WT mice signals for **d** Aβ protein (One-way ANOVA F: (Alborghetti et al. 2024; Blankenship et al. 2024) = 132.5, *p* < 0.0001; Fisher’s LSD, aging WT vs. aging 3xTgAD: t (Blankenship et al. 2024) = 15.07, *P* < 0.0001; aging WT vs. aging AD-TH: t (Blankenship et al. 2024) = 12.88, *P* < 0.0001; aging AD-TH vs. aging 3xTgAD: t(Blankenship et al. 2024) = 2.185, *P* = 0.0567) **e** Hyperphosphorylated tau protein (one-way ANOVA F: (Alborghetti et al. 2024; Blankenship et al. 2024) = 136.0, *P* < 0.0001; Fisher’s LSD, aging WT vs. aging 3xTgAD: t(Blankenship et al. 2024) = 14.58, *P* < 0.0001; aging WT vs. aging AD-TH: t (Blankenship et al. 2024) = 13.97, *P* < 0.0001; aging AD-TH vs. aging 3xTgAD: t (Blankenship et al. 2024) = 0.614, *P* = 0.554) and **f** TH protein (one-way ANOVA F: (Alborghetti et al. 2024; Blankenship et al. 2024) = 7.388, *p* = 0.0126; Fisher’s LSD, aging WT vs. aging 3xTgAD: t(Blankenship et al. 2024) = 2.807, *P* = 0.0205; aging WT vs. aging AD-TH: t (Blankenship et al. 2024) = 3.678, *P* = 0.0051; aging AD-TH vs. aging 3xTgAD: t(Blankenship et al. 2024) = 0.8703, *P* = 0.4068) within CA1 hippocampus. The second-panel set displays confocal images of LC coronal sections showing the immunoreactive signal for **g** Aβ protein (green), **h** Hyperphosphorylated tau protein (green), **i** TH (red), and DAPI (gray) in aging WT (*n* = 4), aging 3xTgAD (*n* = 4), and aging AD-TH (*n* = 4) mice. The ratio of the immunoreactive signal adjusted to the aging WT mice signals for **j** Aβ protein (one-way ANOVA, F: (Alborghetti et al. 2024; Blankenship et al. 2024) = 20.61, *P* = 0.0004; Fisher’s LSD, aging WT vs. aging 3xTgAD: t(Blankenship et al. 2024) = 6.113, *P* = 0.0002; aging WT vs. aging AD-TH: t(Blankenship et al. 2024) = 4.757, *P* = 0.0010; aging AD-TH vs. aging 3xTgAD: t(Blankenship et al. 2024) = 1.359 *P* = 0.207), **k** Hyperphosphorylated tau protein (one-way ANOVA F: (Alborghetti et al. 2024; Blankenship et al. 2024) = 130.5, *P* < 0.0001; Fisher’s LSD, aging WT vs. aging 3xTgAD: t (Blankenship et al. 2024) = 14.70, *P* < 0.0001; aging WT vs. aging AD-TH: t (Blankenship et al. 2024) = 13.14, *P* < 0.0001; aging AD-TH vs. aging 3xTgAD: t (Blankenship et al. 2024) = 1.562, *P* < 0.1527) and **l** TH protein TH, one-way ANOVA, F: (Alborghetti et al. 2024; Blankenship et al. 2024) = 0.4468, *P* = 0.6531) in LC. All results show the mean ratio of immunoreactive signal adjusted to aging WT ± SEM. *: *p* < 0.05 indicated the statistically significant difference compared to aging WT. Scale bar: 50 μm

The AD-TH mouse model was developed to optogenetically activate the catecholaminergic LC projections into the CA1 region of the hippocampus by interbreeding 3xTgAD mice with TH-Cre mice. To determine whether the phenotypic expression of key proteins associated with AD was not altered by the genetic background of the AD-TH mice, we evaluated the Aβ protein and hyperphosphorylated tau protein in both the LC and the CA1 region of the hippocampus. These proteins are known to be overexpressed and accumulate within both the LC and the hippocampus in the context of AD pathology. In our analysis, we also examined TH protein levels in both brain structures to quantify the catecholaminergic neuron somas in the LC and the catecholaminergic terminals in the hippocampus of aging mice. A significant increase in Aβ protein (Fig. 5a and d) and hyperphosphorylated tau protein (Fig. 5b and e) was observed in the aging 3xTgAD and aging AD-TH mice within the CA1 hippocampus compared to aging WT mice. However, no significant differences in the expression of these proteins were found between the two AD mouse models. Additionally, the TH immunoreactive signal in the hippocampus indicated reduced catecholaminergic projections (Fig. 5c and f).

Similarly, Aβ (Fig. 5g and j) and hyperphosphorylated tau (Fig. 5h and k) proteins showed increased immunoreactive signals in the LC of aging 3xTgAD and aging AD-TH mouse models. While both proteins were overexpressed in the LC, no significant difference in the immunoreactive signal for TH was found in the neuron somas of aging WT, aging 3xTgAD, and aging AD-TH mice (Fig. 5i and l). A comparative analysis of the Aβ protein immunoreactive signals between the CA1 hippocampus region and LC indicates a more significant accumulation of Aβ proteins in the hippocampus compared to the LC (LC vs. HIP: t(Bezprozvanny and Mattson 2008) = 8.992, *p* = 0.0001). In contrast, the immunoreactivity of hyperphosphorylated tau protein was significantly higher in the LC than in the hippocampus (LC vs. HIP: t(Bezprozvanny and Mattson 2008) = 3.469, *p* = 0.0133). These findings suggest that the expression and accumulation of Aβ and hyperphosphorylated tau proteins occur differentially in the hippocampus CA1 and the LC Although TH-AD mice demonstrate detectable accumulation of both proteins, Aβ predominantly accumulates in the CA1 region of the hippocampus. In contrast, hyperphosphorylated tau primarily accumulates in the LC of aging AD mice. Furthermore, our phenotypic analysis of catecholaminergic neurons in the LC, along with their projections to the CA1 region of the hippocampus, suggests that the observed reduction in catecholaminergic neurotransmitter levels in the hippocampus is due to a decline in catecholaminergic projections rather than a loss of catecholaminergic neurons in the LC.

## Discussion

The progression of AD involves ongoing damage to neuronal function in both the cortical and hippocampal regions. Notably, AD mouse models show significant degeneration of catecholaminergic terminals in the hippocampus by around 12 months of age. This initial damage is followed by neuronal degeneration in the LC and VTA at 18 and 24 months, respectively. The progressive decline in catecholaminergic activity is primarily attributed to the deposition of Aβ peptide (Grudzien et al. 2007; Liu et al. 2008; Moreno-Castilla et al. 2016). This decline is significant because it is associated with the cognitive deficits commonly observed in AD (Braak and Del Tredici 2016; Hansen 2017; Dahl et al. 2023). Recent research has revealed a denser projection of catecholaminergic neurons from the LC to the CA1 region of the hippocampus, which is crucial for memory processes. In contrast, the VTA mainly influences the ventral regions of the hippocampus (Titulaer et al. 2021; Wilmot et al. 2024). Furthermore, catecholaminergic projections from the LC to the dorsal hippocampus significantly modulate synaptic plasticity and the cellular mechanisms involved in encoding spatial information within the hippocampus (Lemon and Manahan-Vaughan 2012; Mravec et al. 2014; Hansen and Manahan-Vaughan 2015; Kaufman et al. 2020; Gálvez-Márquez et al. 2022). These findings highlight the critical role of catecholaminergic neuromodulation from the LC to the hippocampus in regulating memory processes. Additionally, a notable reduction in catecholaminergic projections from the LC to the CA1 region may contribute to the retrieval deficits observed in AD mouse models. This decrease in catecholaminergic projections in the CA1 region has also been observed in AD patients and other AD mouse models (Reinikainen et al. 1988; Chen et al. 2022).

### Optogenetic activation of the LC-CA1 projections improves the catecholaminergic neurotransmission within the hippocampus of AD-TH mice

We observed a significant decrease in the baseline levels of DA and NA in the CA1 area of the hippocampus in 3xTg-AD and AD-TH aging mice. The reduced levels of these neurotransmitters in the hippocampus of AD patients are believed to contribute to the cognitive dysregulation that underlies AD symptoms (Lyness et al. 2003; Francis et al. 2012; Hagena et al. 2016). Our findings indicate that the decrease in DA and NA levels in the CA1 region is associated with a reduction in hippocampal CA1 catecholaminergic projections from the LC rather than a loss of LC catecholaminergic neurons, at least in 12-month-old AD mice. We also showed that optogenetic stimulation of the CA1 hippocampal catecholaminergic projections from the LC is enough to restore hippocampal catecholaminergic levels. The LC is a crucial brain region where abnormal, hyperphosphorylated tau protein accumulates in individuals with AD (Mather and Harley 2016; Beardmore et al. 2021). In both of our transgenic mouse models, the levels of hyperphosphorylated tau protein in the LC align with Braak staging and have been detected prior to the onset of tau pathology in the hippocampus (Grudzien et al. 2007; Braak and Del Tredici 2016). Although we did not observe a decrease in TH + neurons in the LC of the aging AD mice, the accumulation of abnormal tau protein could still lead to degeneration of catecholaminergic projections. This degeneration may result in the reduced basal release of DA and NA in the hippocampus, which is necessary for retrieving spatial memory (Moreno-Castilla et al. 2016; Gálvez-Márquez et al. 2022).

Additionally, the Aβ peptide plays a neuromodulatory role in regulating synaptic communication and neurotransmitter release (Karisetty et al. 2020), mediated through direct and indirect interactions with presynaptic proteins that control neurotransmitter vesicle release (Abramov et al. 2009; Russell et al. 2012; Gulisano et al. 2019). However, the accumulation of Aβ peptide has a detrimental effect on neurotransmitter release. The combination of diminished LC catecholaminergic projections and the potential adverse consequences of Aβ peptide accumulation may contribute to the diminished catecholaminergic levels observed here, thereby impeding the hippocampal synaptic efficiency and negatively impacting the retrieval of spatial memories in AD mice. Nevertheless, we show that the pathological low level of catecholamines in the hippocampus can be reversed through the optogenetic stimulation of the LC catecholaminergic projections into the CA1 hippocampal region in the AD-TH mice.

### Optogenetic stimulation of the LC-CA1 projections reverses Spatial memory retrieval deficiencies in AD mice

Research on AD in mouse models suggests that the primary challenge lies in retrieving memories rather than storing them (Billings et al. 2005; Roy et al. 2016; Jura et al. 2019; Small and Cochrane 2020; Bostancıklıoğlu 2020). This difference is likely due to impaired activation of the memory engrams required for retrieving spatial memory (Roy et al. 2016; Perusini et al. 2017). Our findings support this hypothesis. We observed that young AD mice can store and retrieve spatial information in both spatial memory paradigms. However, 3xTgAD and AD-TH aging mice struggle to retrieve spatial memory during LTM. We found that optogenetic stimulation of the catecholaminergic projections from the LC-CA1 of the hippocampus, administered before LTM, significantly improved spatial memory retrieval in the aging AD-TH mouse model. These results suggest that AD-TH mice can form and consolidate hippocampal-dependent memories but encounter challenges in retrieving these memories. The optogenetic stimulation enhances the recognition of a novel object’s position, which is linked to the release of DA and NA. Previous studies indicate that DA is responsible for driving the response to novelty (Kempadoo et al., 2016; Takeuchi et al., 2016), while NA is associated with the saliency of novel objects, influenced by LC terminals in the hippocampus (Gálvez-Márquez et al., 2022). Additionally, the optogenetic inhibition of LC catecholaminergic projections into the CA1 region of the hippocampus hinders the retrieval of spatial memory (Gálvez-Márquez et al., 2022). This finding underscores the significance of catecholaminergic activity from the LC in effectively retrieving spatial memories in the hippocampus.

Additionally, administering a DA D1-like receptor antagonist or a beta-adrenergic receptor antagonist before an LTM session in the CA1 region of the hippocampus impairs spatial memory retrieval (Gálvez-Márquez et al. 2022). In this context, administering NA precursors or DA agonists has been shown to reduce the toxic effects of Aβ and improve performance in spatial memory tasks (Himeno et al. 2011; Kalinin et al. 2012; Gutiérrez et al. 2022). Research also indicates that blocking DA reuptake in the cortex of AD transgenic mice can alleviate memory recognition impairment and enhance DA activity (Guzmán-Ramos et al. 2012, 2022; Moreno-Castilla et al. 2016). Together, these studies emphasize that catecholaminergic activity is closely related to AD dysfunction, representing a potential neurochemical target for pharmacological treatment. Consequently, using DA agonists, rotigotine, the DA precursor L-DOPA or the monoamine oxidase-B inhibitor selegiline has enhanced synaptic plasticity and improved memory deficits in experimental AD models and humans (Koch et al. 2014; Dahl et al. 2023). Further investigation is necessary to identify the specific catecholaminergic receptors involved in memory retrieval and to address the limitations of optogenetic protocols, such as the temporal resolution of photoinduced responses and local photostimulation. However, our findings suggest that stimulating catecholaminergic pathways in the brain could help restore memory retrieval in AD murine models. Changes in the firing of the LC may influence saliency and novelty, which in turn facilitates spatial memory retrieval (Hagena and Manahan-Vaughan, 2024). Although the parameters used for optogenetic stimulation differ from natural LC responses, where bursts of activity consist of 2 to 4 spikes at frequencies of 10 and 25 Hz (Clayton et al., 2004; Takeuchi et al., 2016), they still promote catecholamine release in the hippocampus, potentially leading to therapeutic effects. Notably, this study is the first to highlight the critical and sufficient role of LC catecholaminergic activity in the hippocampus for improving memory retrieval in AD.

### Optogenetic activation of the LC-CA1 projections restores synaptic plasticity in aging AD-TH mice

Our experimental approach suggests that enhancing spatial memory retrieval in aging AD mice may be associated with promoting hippocampal neural plasticity. This improvement could result from restoring catecholaminergic levels through optogenetic stimulation of the LC catecholaminergic projections within the CA1 region of the hippocampus. It is well known that DA and NA transient bursts (phasic activity) occur in response to rewarding or novel stimuli, promoting LTM (Harley 2004). Unlike the aged AD mice, which cannot induce LTP in response to HFS, the groups of young WT, aged WT, and young AD mice exhibit normal synaptic plasticity. These findings support the notion that there is an age-dependent progression of pathology in AD models, resulting in a gradual decline in synaptic plasticity over time.

Catecholamines play a crucial role in modulating the molecular and cellular mechanisms that contribute to synaptic plasticity through their specific receptors (Twarkowski and Manahan-Vaughan 2016; Madadi Asl et al. 2019). A reduction in innervation can lead to disinhibition and increased excitability in hippocampal areas, a phenomenon commonly observed in the early stages of AD (Goettemoeller et al. 2024). NA interacts with α1, α2, and β adrenergic G-protein coupled receptors (GPCRs), activating Gs subunits and cyclic adenosine (cAMP) pathways, which significantly influence synaptic plasticity (Marzo et al. 2009; Maity et al. 2020, 2022). Additionally, NA can induce hyperpolarization in cortical neurons (Wong et al. 2023) and enhance LTP in the CA1 region by promoting protein kinase A (PKA) pathways (Gelinas et al. 2008) and activating guanine nucleotide exchange proteins through cAMP (Brandwein and Nguyen 2019).

On the other hand, DA influences synaptic function through D1-like and D2-like GPCRs, which promote LTP and LTD in the hippocampus, respectively (Caragea and Manahan-Vaughan 2021; Kim et al. 2022). While ionotropic receptors mediate rapid neuronal responses through the immediate flow of ions across the cell membrane, GPCRs trigger a slower activation of intracellular signaling cascades. This process indirectly affects the release of ions and neurotransmitters, ultimately influencing gene expression and synaptic plasticity (Betke et al. 2012). Although hippocampal CA1 synaptic plasticity in the AD-TH mice was reinstated by optogenetic stimulation of the LC catecholaminergic projections, we observed a slow onset of LTP induction. As mentioned earlier, this slow onset may be attributed to the delayed response elicited by the GPCRs (Greengard 2001; Wong et al. 2023; Tse et al. 2023).

Recent studies suggest that synaptic plasticity in the hippocampus can be influenced by optogenetic stimulation of catecholaminergic projections in healthy mice (Takeuchi et al. 2016; Kempadoo et al. 2016; Gálvez-Márquez et al. 2022). The activity of catecholamines from the LC highlights the importance of neural communication within the hippocampus (Hansen 2017). The optogenetic inhibition of hippocampal catecholamine projections from the LC can alter the threshold for transitioning from LTP to LTD following high-frequency stimulation (Gálvez-Márquez et al. 2022). This change may be linked to decreased levels of DA and NA. Additionally, DA depletion caused by the Aβ peptide has been shown to impair synaptic plasticity, converting HFS-induced LTP into LTD in the dorsal hippocampus (Mayordomo-Cava et al. 2020) and cortical circuits (Moreno-Castilla et al. 2016). Similarly, the co-administration of DA and beta-adrenergic receptor antagonists can induce LTD following high-frequency stimulation (Gálvez-Márquez et al. 2022). Interestingly, we have found that increased cortical DA activity can reverse LTD induced by Aβ1–42 oligomers back into LTP (Guzmán-Ramos et al. 2012, 2022; Moreno-Castilla et al. 2016). These results align with the observed increase in catecholamines when photostimulation of the LC-CA1 terminals leads to improved memory and a shift from LTD to LTP in the hippocampal CA1 region. Our experimental approach suggests that stimulating the catecholaminergic system is crucial to retrieve LTM by enhancing synaptic plasticity mechanisms, and recent works showed that forms of LTP and LTD in the LC enable the acquisition and retention of spatial memories (Hagena and Manahan-Vaughan 2024). These changes are essential for activating and triggering the memory engram, enabling AD-TH mice to retrieve previously acquired spatial information.

## Conclusion

The exact causes and underlying mechanisms of AD are not yet fully understood. However, it is essential to develop strategies to improve the memory deficits associated with AD for clinical treatments. Some experimental approaches suggest that stimulating the catecholaminergic system through pharmacological methods (Koch et al. 2014; Shaikh et al. 2023) or repeated exposure to novel stimuli could be critical for cognitive improvement (Velázquez-Delgado et al. 2024). These strategies emphasize the influence of lifestyle and environmental factors on AD and their potential to alleviate symptoms. Our results align with these findings and suggest that therapies targeting the projections from the LC to the hippocampus, and probably the cortex, could enhance cognitive function in patients with mild cognitive impairment due to early AD. These results highlight the significance of targeting LC catecholaminergic neurons as potential therapeutic targets to address cognitive deficits in AD patients through targeted stimulation.

## Supplementary Information

Below is the link to the electronic supplementary material.

Supplementary file 1 (DOC 568KB)

## Data Availability

Analyzed data will be made available upon reasonable request to the corresponding author.
