# Supplementary material for: Photostimulation of locus coeruleus CA1 catecholaminergic terminals reversed Spatial memory impairment in an alzheimer’s disease mouse model
Source: Psychopharmacology (Berl). 2025 Sep 8;243(4):831–50. doi: 10.1007/s00213-025-06885-w (PMC13035695; doi:10.1007/s00213-025-06885-w)
Supplement: Supplementary file 1 — (DOC 568KB) [file 213_2025_6885_MOESM1_ESM.doc]

**Supplementary Information for**

**Photostimulation of Locus Coeruleus CA1 catecholaminergic terminals reversed spatial memory impairment in an Alzheimer's disease mouse model.**

Donovan K. Gálvez-Márquez, Oscar Urrego-Morales, Luis F. Rodríguez-Durán, and Federico Bermúdez Rattoni.

**This PDF file includes:**

Supplementary Fig. 1 OLM in the young AD-TH mouse model

Supplementary Fig. 2 Spatial memory in the young AD-TH mouse model

Supplementary Fig. 3 Long-term plasticity in the young AD-TH mouse model

**Supplementary Fig. 1** **Aging impairs the OLM in the AD-TH mouse model.** **a** OLM Protocol. **b** Mean recognition index of two objects during the acquisition phase was analyzed using a two-way ANOVA. The results showed no significant difference between the objects (F(1, 106) = 1.031, P = 0.3122). Additionally, LTM results were observed for young groups: WT (n = 8), 3xTgAD (n = 11), and AD-TH (n = 5). **c** Mean recognition index of two objects during the LTM phase, two-way ANOVA indicated a significant difference between the objects (F(1, 92) = 42.20, P < 0.0001). Fisher’s LSD post-hoc tests revealed the following results: WT (young) compared to NL with FL (t(92) = 8.697, P < 0.0001); 3xTgAD (young) (t(92) = 3.953, P = 0.0002); AD-TH (young) (t(92) = 3.842, P = 0.0002). **d** Total exploration time of the two objects (first bar corresponding to acquisition 1, second bar corresponding to acquisition 2 and third bar corresponding to LTM), and was assessed using a two-way ANOVA, which showed no significant difference between days (F(2, 86) = 1.221, P = 0.2999) or between groups (F(5, 43) = 1.593, P = 0.1825). All results showed mean ± SEM. *: P < 0.05. s: seconds.

**Supplementary Fig. 2. Spatial memory in the young AD-TH mouse model**. **a** MWM protocol. **b** Latency time to the platform during acquisition days for WT (young) n = 8, 3xTgAD (young) n = 10, and  AD-TH (young) n = 6. The results show the mean latency time during the four days of acquisition sessions. All mouse groups demonstrated improved performance in reaching the hidden platform over time, with no statistically significant differences observed between the groups (Repeated measures two-way ANOVA: groups F(5, 43) = 0.6843, P = 0.6379; time F(3, 129) = 129.3, P < 0.0001). Post hoc analysis using Fisher's LSD revealed the following comparisons: WT (young) latency time acquisition 1^sh^ day vs. 4^th^ day: t(129) = 8.809, P < 0.0001; 3xTgAD (young) latency time acquisition 1^sh^ day vs. 4^th^ day: t(129) = 8.182, P < 0.0001; and AD-TH (young) latency time acquisition 1^sh^ day vs. 4^th^ day: t(129) = 7.733, P < 0.0001; During the LTM test, memory retrieval was evaluated. **c** The number of crosses to the platform area. **d** Percent of time spent in the target quadrant. **e** Latency time to the platform area **f** Swimming speed for the mice groups. No differences were observed between young and aging. All results showed the mean ± SEM.

**Supplementary Fig. 3.** **Long-term plasticity in the young AD-TH mouse model. a** The schematic illustrates the placement of recording and stimulation electrodes in the Schaffer collaterals of the dorsal hippocampus. **b** The high-frequency stimulation protocol consists of three trains of 100 pulses at 100 Hz. **c** and **d** The responses of the percentage of fEPSP are shown for young groups **c** WT, n = 5), **d** 3xTgAD (n = 6), and **e** AD-TH (n = 5). **f** A representative graph depicts the percentage of fEPSP slope over the last fifteen minutes (represented by the gray line). The statistical analysis was a one-way ANOVA, which yielded F(5,35) = 7.795, P < 0.0001. The results of the Holm-Sidak post-hoc tests are as follows: WT (young) vs. 3xTgAD (young): t(35) = 0.1105, P = 0.9987; WT (young) vs. AD-TH (young): t(35) = 0.4525, P = 0.9987;. All results represent the mean percentage of fEPSP slope at baseline ± SEM.
